# Pediatric Emergency Medicine Podcasts: Current Status

**DOI:** 10.7759/cureus.28384

**Published:** 2022-08-25

**Authors:** Ricardo Hernandez, Yaron Ivan, Eva Esperanza, Andrew Little

**Affiliations:** 1 Emergency Medicine, AdventHealth East Orlando, Orlando, USA; 2 Pediatric Emergency Medicine, AdventHealth Orlando, Orlando, USA; 3 Emergency Medicine, University of Central Florida, Orlando, USA

**Keywords:** pediatric emergency medicine podcasts, pediatrics, emergency medicine training, emergency medicine resident, emergency medicine, podcasts, pediatric emergency medicine

## Abstract

Background: Podcasting has become a primary delivery model for medical content among various specialties. Although this model is still growing, it has become an essential tool for many learners, educators, and institutions. Because of this rapid growth, there is an unknown availability of podcasts for each specialty.

Objectives: This paper aimed to evaluate the podcasts currently available in the subspecialty of pediatric emergency medicine (PEM).

Methods: The investigators sought to evaluate the prevalence of PEM podcasts from the end-user's (medical students, residents, etc.) perspective. This was completed by performing a simple internet search using the term “podcasts in pediatric emergency medicine.” Using Google Search, the first 50 results were analyzed.

Results: For PEM, there were only eight podcasts found, five of which were active.

Conclusion: PEM podcasts in comparison to other specialties are currently underrepresented and lacking in this important resource. The authors call on PEM physicians, educators, and organizations to consider creating content in this educational space.

## Introduction

Over the past decade, podcasting has become a primary delivery model for medical content among various specialties [1]. During that time, the breadth of medical podcasts and the depth of content has gone through tremendous growth [2]. With the increasing number of podcasts available, there has also been some research showing that many podcasts are unsustainable or suffer from a phenomenon known as “pod fade,” where a podcast is produced and for any reason stops releasing content and/or episodes [3].

Numerous studies have demonstrated that podcasts in medical education are valuable among learners, educators, and institutions [4,5]. Many researchers have evaluated the use of medical podcasts in education and have outlined best practices using information from the end-user's perspective [4]. One study found that medical students who listened to a series of podcasts on various topics related to their coursework had better grades than those who did not listen to the podcasts [6]. In another study, residents who listened to medical podcast episodes during their commute demonstrated increased knowledge and reported that they would be likely to continue listening to podcasts as part of their education [7]. Also, a previous study shows that if pediatric content is created, it will be consumed by medical professionals [8].

While many authors have looked at how to best use podcasts in medical education and the role they play in clinical care, few have attempted to catalog the availability of medical content in various specialties. One previous study outlined podcasts available in mainstream specialties, while another evaluated the major subspecialties within emergency medicine that did not include pediatric emergency medicine (PEM) [9]. This paper aimed to evaluate the podcasts currently available in the subspecialty of PEM. Specifically, we attempted to list the known podcasts, indicate their hosts or institution, and provide an overview of their content.

## Materials and methods

This was a Google-based, investigational study of medical podcasts by specialty undertaken by all authors from June through August 2021. Search terms included “podcasts in pediatric emergency medicine.” After performing this search, we set out to find 50 podcasts on PEM. The search was conducted using the Google search engine and was carried through the first five search pages. Fifty sites were reviewed. The first step during the review was determining whether the site represented an actual PEM podcast. Exclusion criteria included third-party podcasting websites and websites where the information in question was not available. Also, individual episodes about pediatrics in emergency medicine, but not part of a “pediatric emergency medicine podcast,” were not included in our data. All other podcasts were included. Once a podcast was identified, the first determination was “active” versus “inactive.” For this definition, we deemed any podcast that had not released an episode within six months before the search as being “inactive.” For each podcast, additional data were collected, including the number of available episodes, podcast name, release schedule, average run time, and current host(s).

## Results

Only eight podcasts were found to be exclusive PEM podcasts (see Table 1). Among those, only five were found to be currently active. The first search on the list was PEM Currents, hosted by Dr. Brad Sobolewski, the most active podcast of all, with 77 episodes averaging 13 minutes per episode. Regarding frequency, it was sporadic, with a timelapse between episodes ranging from nine to 60 days. Second on the list was EMPEM hosted by Dr. Colin Parker on January 30, 2010. It is currently inactive with the last episode posted on June 9, 2014, with 49 episodes. Third on the list was PEMED, hosted by Dr. Andrew Sloas on July 1, 2011. It is currently inactive, with the last episode posted on July 12, 2015, with a total of 31 episodes. Fourth on the list was the Pediatric Emergency Playbook, hosted by Dr. Tim Horeczko, which is currently active with a total of 66 episodes averaging 41 minutes per episode, and a new episode published on the first of every month. Fifth on the list was Paediatric Emergencies, hosted by Dr. Christopher Flannigan on April 19, 2015. It is currently inactive, with the last episode posted on June 13, 2019, with 26 episodes. Pediatric Emergency Medicine was the sixth on the list and is hosted by Dr. Robert Belfer. It had the least number of episodes out of all the podcasts, with only two episodes lasting 43 minutes, but it is still considered an active podcast as the episodes were released within the past six months. The episodes are released monthly. The second most active podcast was Ped Reviews and Perspectives, hosted by a rotating group of doctors. It has 70 episodes released monthly with an average duration of 180 minutes. The final podcast was PEM Rules, hosted by Dr. Yaron Ivan. The podcast has four episodes, which were released bi-monthly with an average length of 10 minutes. It was worth pointing out that over 25 emergency medicine (EM) podcasts had PEM content (or episodes).

**Table 1:** Current pediatric podcasts

Podcast name	Release schedule (monthly, bi-monthly, sporadic)	Average length (minutes) (average of 5 most recent podcasts)	Hosted by	Website	Active (an episode in the last 6 months)
PEM Currents	Sporadic	13	Brad Sobolewski	http://pemcincinnati.com/blog/	Yes
EMPEM	Sporadic	35	Colin Parker	http://empem.org/	No
PEMED	Sporadic	51	Andrew Sloas	http://www.pemed.org/	No
Pediatric Emergency Playbook	Monthly	41	Tim Horeczko	http://pemplaybook.org/	Yes
Paediatric Emergencies	Sporadic	25	Christopher Flannigan	https://www.paediatricemergencies.com/podcasts/	No
Pediatric Emergency Medicine	Monthly	43	Robert Belfer	https://www.chop.edu/pempodcast	Yes
Ped Reviews and Perspectives	Monthly	180	Rotating	https://www.hippoed.com/peds/rap/	Yes
PEM Rules	Bi-monthly	10	Yaron Ivan	https://pemrules.com/	Yes

## Discussion

Reviewing the available data shows that podcasts play a role in undergraduate and graduate medical education [10-12]. Also, when used by learners from other specialties, podcasts shape their medical knowledge and alter their clinical practice [13]. It has also been shown that medical professionals prefer podcasts to other forms of media in education delivery, specifically, podcasts over video recordings, printed media, and in-person education [14,15]. These factors should be taken into consideration when evaluating our findings.

Our study indicates that, currently, there is a very small number of active PEM podcasts. This is especially true when comparing the number of active PEM podcasts to the number of active general EM podcasts, which are currently over 30 and growing [2]. But these findings align with the numbers and length of other EM specialty podcasts currently produced and available [9]. Given the expanding field of PEM, coupled with the fact that PEM plays a decent role in most EM residencies, we believe that there is a growing need for more PEM education, some of which can be provided via podcasts. In addition, many residencies such as family medicine and pediatrics have PEM as part of their core rotation curriculum, and we believe PEM education can benefit their trainees as well.

This study is not without limitations. Based solely on internet searches to obtain our information, several additional podcasts may be available. But due to not having an official website or optimization parameters, their site may not have been found in our 50 site search parameters. There also could be several podcasts that use paywall or subscription services that we could not evaluate or podcasts created for “internal use” that are unavailable to the public. Opportunities for further research include evaluating learner utilization of podcasts, as it is unknown if the number of podcasts and episodes available per specialty relates to the number of listeners.

## Conclusions

With PEM being a growing field within EM, we believe there is a need for more PEM education for medical students, residents, fellows, and attendings. We believe podcasts can serve as a platform fulfilling part of that need, as they have in other undergraduate and graduate medical education, and with learners from various specialties. Our study indicates that currently there are very few active PEM podcasts and with a growing need for more to be created to sustain the educational needs as already outlined. We call on PEM and other EM providers to consider creating and disseminating PEM content in the form of new PEM podcasts.
